# Supplementary figures and images for: Alpine treeline ecotone stasis in the face of recent climate change and disturbance by fire
Source: PLoS One. 2020 Apr 10;15(4):e0231339. doi: 10.1371/journal.pone.0231339 (PMC7147793; doi:10.1371/journal.pone.0231339)

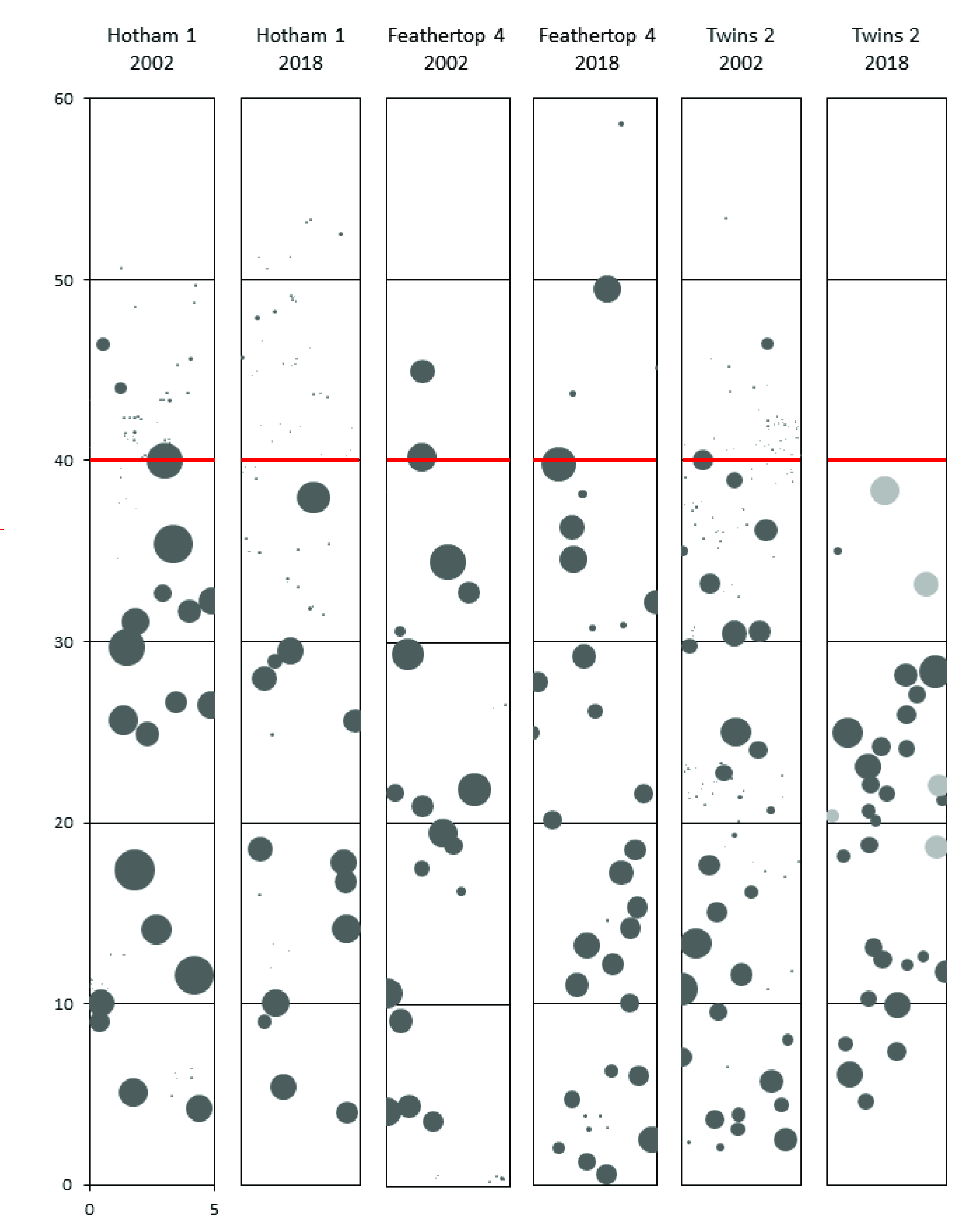

Supplement: S1 Fig — X and Y axes indicate exact meter locations across the transect. Circle size indicates basal circumference in relative proportions to the X and Y axes. Grey = dead individuals. Black = live individuals. Treeline is represented by the red line at y = 40m, y<40 within the woodland, y>40 above treeline. (TIF) [file pone.0231339.s001.tif]
