# Supplementary material for: Alpine treeline ecotone stasis in the face of recent climate change and disturbance by fire
Source: PLoS One. 2020 Apr 10;15(4):e0231339. doi: 10.1371/journal.pone.0231339 (PMC7147793; doi:10.1371/journal.pone.0231339)
Supplement: S1 Table — (DOCX) [file pone.0231339.s002.docx]

| **Site** | **Transect** | **Transect Length (m)** | **Latitude, Longitude** | **Aspect** | **Elevation (m a.s.l)** | **Stock grazing removed** | **Recent fire history** |
| --- | --- | --- | --- | --- | --- | --- | --- |
| Mount Hotham | 1 | 41.80 | -36.97785379, 147.1242343 | W | 1796 | 1958 | N/A |
|  | 2 | 59.48 | -36.97524903, 147.1266789 | W | 1830 | 1958 | N/A |
|  | 3 | 45.31 | -36.97644968, 147.1254575 | NW | 1795 | 1958 | N/A |
|  | 4 | 74.02 | -36.97892085, 147.1247683 | SW | 1792 | 1958 | N/A |
|  | 5 | 86.80 | -36.97884944, 147.1246779 | SW | 1794 | 1958 | N/A |
| Mount McKay | 1 | 82.95 | -36.87497290, 147.2393232 | NW | 1770 | 1981 | 2003 |
|  | 2 | 46.16 | -36.87724029, 147.2410168 | S | 1710 | 1981 | 2003 |
|  | 3 | 43.40 | -36.87746503, 147.2379679 | S | 1684 | 1981 | 2003 |
|  | 4 | 52.27 | -36.87462649, 147.2407710 | NW | 1799 | 1981 | 2003 |
| Mount Feathertop | 1 | 42.00 | -36.88759302, 147.1391903 | N | 1789 | 1958 | 2003 |
|  | 2 | 54.80 | -36.88763299, 147.1390171 | NW | 1785 | 1958 | 2003 |
|  | 3 | 40.60 | -36.89706216, 147.1338984 | W | 1881 | 1958 | 2003 |
|  | 4 | 45.15 | -36.89755056, 147.1327111 | W | 1776 | 1958 | 2003 |
| The Twins | 1 | 40.00 | -37.02337328, 147.0592530 | N | 1657 | 2003 | 2003, 2013 |
|  | 2 | 40.00 | -37.02341110, 147.0599431 | N | 1649 | 2003 | 2003, 2013 |
|  | 3 | 42.85 | -37.02392545, 147.0616333 | N | 1659 | 2003 | 2003, 2013 |
|  | 4 | 40.00 | -37.02306921, 147.0567063 | W | 1677 | 2003 | 2003, 2013 |
|  | 5 | 40.00 | -37.02291647, 147.0568966 | W | 1681 | 2003 | 2003, 2013 |
|  | 6 | 40.45 | -37.02308836, 147.0565635 | W | 1673 | 2003 | 2003, 2013 |
