# Supplementary material for: Alpine treeline ecotone stasis in the face of recent climate change and disturbance by fire
Source: PLoS One. 2020 Apr 10;15(4):e0231339. doi: 10.1371/journal.pone.0231339 (PMC7147793; doi:10.1371/journal.pone.0231339)
Supplement: Attachment — Submitted filename: Response to Reviewers.docx [file pone.0231339.s003.docx]

**Response to Reviewers**

**1. Is the manuscript technically sound, and do the data support the conclusions?**

The manuscript must describe a technically sound piece of scientific research with data that supports the conclusions. Experiments must have been conducted rigorously, with appropriate controls, replication, and sample sizes. The conclusions must be drawn appropriately based on the data presented.

Reviewer #1: Yes

Reviewer #2: Yes

**2. Has the statistical analysis been performed appropriately and rigorously?**

Reviewer #1: Yes

Reviewer #2: Yes

**3. Have the authors made all data underlying the findings in their manuscript fully available?**

The [PLOS Data policy](http://www.plosone.org/static/policies.action#sharing) requires authors to make all data underlying the findings described in their manuscript fully available without restriction, with rare exception (please refer to the Data Availability Statement in the manuscript PDF file). The data should be provided as part of the manuscript or its supporting information, or deposited to a public repository. For example, in addition to summary statistics, the data points behind means, medians and variance measures should be available. If there are restrictions on publicly sharing data—e.g. participant privacy or use of data from a third party—those must be specified.

Reviewer #1: Yes

Reviewer #2: Yes

**4. Is the manuscript presented in an intelligible fashion and written in standard English?**

PLOS ONE does not copyedit accepted manuscripts, so the language in submitted articles must be clear, correct, and unambiguous. Any typographical or grammatical errors should be corrected at revision, so please note any specific errors here.

Reviewer #1: Yes

Reviewer #2: Yes

**5. Review Comments to the Author**

Please use the space provided to explain your answers to the questions above. You may also include additional comments for the author, including concerns about dual publication, research ethics, or publication ethics. (Please upload your review as an attachment if it exceeds 20,000 characters)

**Reviewer #1:** Review Naccarella et al.

Alpine treeline ecotone stasis in the face of recent climate change and disturbance by fire.

**General comments:**
Naccarella and colleagues revisited vegetation surveys from different locations at the alpine treeline in south-eastern Australia, to assess the impact of forest fires on upslope movement of treelines and forest stand structure. They could clearly show that in areas with an unusually high fire frequency, sapling establishment was reduced both within the subalpine forests and above treeline. They conclude that fire has the potential to impede the upslope movement of treelines in response to a warming climate.

I’ve greatly enjoyed reading this well-written and concise manuscript. The authors make a very convincing case for fire as an important secondary control of treeline dynamics. This is an important finding that could explain the lack of evidence of an upward shift of mountain forests in some parts of the world, and has implications for assessing future vegetation dynamics under climate change. Of course, it is a pity that the exact transects could not have been reproduced between sampling years. Nevertheless, the authors can show clear trends in response to fire on the local stand-scale. Even though I mostly agree with the interpretation and conclusions of the authors I have two remarks:

- To use the presence of seedlings/saplings above treeline to infer a response to warming temperatures is a bit tricky. As you state yourself, seedlings/saplings are sheltered and not exposed to the atmosphere, and therefore a normal occurrence above treelines also without any warming trend. To directly infer a climate-driven upward shift of treeline position as hypothesized in Fig. 1, you would need to compare actual treelines. An upward shift of saplings as presented in Fig. 2a is a potential indication at best, especially given the very small increase of 5 m (!), which is well below what you would expect from the climate data (0.4°C warming since 1992, so about 50 m using standard lapse rates). The raw number of seedlings (as in Fig. 2c) is no indication at all. However, I fully agree with your conclusion that by increasing sapling mortality (effectively removing any saplings above treeline), fire has the potential to impede the upslope movement of subalpine forests. If only temporarily or permanently depends on future fire frequencies and a potential shift in fire regimes.

Thank you for raising this concern. Firstly our investigation of treelines in the Victorian Alps began with the idea that given the 0.4°C of warming we should theoretically be seeing a substantial increase in the position of sapling trees (i.e. above the well-established tree distribution), if overall temperature was the key driver of treeline position. Given we were not seeing this extent of treeline advance we then considered alternative controls on treeline positions. Our investigation then became driven by bushfires due to the occurrence of 3 landscapes scale fires within 10 years within the Victorian Alps, well outside historic fire intervals. As such, we attempted to present this manuscript in terms of raising the question of bushfires as a key influence on treeline positions, a factor which will become more influential into the future with increases in fire frequency and intensity predicted. As we did not observe dramatic shifts in treeline positions as predicted with temperature, we investigated changes in treeline dynamics and woodland structure. We did this by comparing saplings numbers (we did not observe any seedlings <1 year old with cotyledons still visible) above treeline as indicators of changing conditions above treeline. We agree that the small increase in saplings does not indicate a current shift in treeline positon; however, we attempt to say that the presence of saplings above treeline could indicate the potential for future treeline advance, if conditions remain favourable for establishment and growth above treeline. Given *E. pauciflora* are very long-lived (300+ years) it is not necessarily unexpected that there appears to be long time lags between climate and treelines in the Victorian Alps.

- Forest fires normally depend on two factors: climate and fuel. Without enough fuel there is no fire. So, I was wondering what the fuel loads above or at treeline look like and how far fires can penetrate into the alpine vegetation as brush (grass?) fires. Could you comment on this? I agree that since sapling establishment is restricted to areas close to the treeline (10 m) fire would still kill of most saplings (as you show) but what about single individuals located farther up? Would they still be affected? And with temperature projections of up to +3.8°C could single individuals establish above the zone affected by forest fires, if seeds somehow get there?

Thank you for raising this. Australian alpine areas are vegetated and often comprise grassland, heathland and herbfield plant communities. Fuel loads remain high even above treeline and fires are likely to burn in the alpine zone, as well as in the woodland, since the shrub component of the vegetation is highly flammable and composed of typical Australian families that have volatile oils in their leaves (*Proteaceae*, *Rutaceae*, *Lamiaceae*, *Asteraceae*). Fraser *et al.* (2016) calculated fuel loads in the Victorian Alps and found long unburnt closed heathland to have the highest fuel loads (6.6 kgm^-2^) followed by open heathland (2.4 kgm^-2^) then grassland (1.6 kgm^-2^). In terms of fire affecting individual trees above treeline, given the fuel loads, it would be possible to think that the increase in trees above treeline would increase fire activity and severity in these areas, with fire spotting between clumps of trees. Additionally, if trees have established above treeline this could also create a microclimate for shrubs, which also increase flammability. Similarly, greater shrub cover, due to warmer conditions, at lower elevations below treeline is predicted to increase fuel loads and flammability (Camac *et al*. 2017).

I recommend the article for publication in PLOSone after addressing these remarks and some minor comments below:

L. 158: How are “large adult trees” defined? >2 m height? Basal diameter >25 cm?

Thank you for raising this. Large adult trees were defined as >3 m height and >25 cm basal girth. We have now added a sentence to address this in the methods, and added a sentence to define our use of seedling, sapling and adult.

L. 237: How was the age of saplings estimated? Just by diameter? Because in Fig. 3 the oldest “saplings” have supposedly established in 1939, which means that they are not technically saplings (as in “young trees”) anymore, but rather quite old trees that were growing very slowly due to the harsh conditions above treeline.

Thank you, we have now amended this section. With this data we hoped to gain an understanding of the longevity of individuals above treeline and to indicate whether there had been a spike in establishment in recent years, following warming climates and bushfires. As such we measured the diameter and calculated age of establishment of all individuals (saplings and adults) above treeline in our surveys. Figure 3 states the estimated year of establishment of individuals above treeline. This is correct as we measured the diameter of all individuals (saplings and adults) above treeline, from which the age was calculated based on the methods of Rumpff *et al*. (2009).There was an error in line 237 which should state “Estimates of the age of individuals above treeline in 2002 and 2018 indicate that most had established after 1995 and then again after 2012 (Fig 3).” This has been adjusted.

L. 240-241: This is a bit confusing, since in the previous sentence you mention that “…mortality above treeline was high.” And here you state that “Tree mortality was low…”. I guess you talk about actual dead individuals found, whereas in the previous sentence you base your interpretation on the difference between the survey period, which clearly also includes seedlings. I suggest rephrasing to make this distinction clear.

Thank you for raising this, we have now rephrased these sentences.

L.297-310: I fully agree, what you observe here, a large number of seedlings/saplings above the treeline with very low to zero long-term survival due to microclimatic conditions (ground vs. athmosphere) is a very common feature of treeline ecosystems around the world. But this is also why it is not a good idea to use seedling/sapling establishment above treelines as an indicator of an upslope movement of treelines in response to warming temperatures.

Thank you for raising this, however we respectfully disagree. We set out to investigate changes in seedling/sapling numbers as a descriptor of treeline dynamics, not as a definitive indicator of treeline movement. Through observing changes in sapling numbers above treeline we investigated changes in treeline dynamics and thus the potential for treeline advance in the future, if individuals persist above treeline. Despite regional warming we saw very little establishment of saplings above treeline and hence, the treeline is remaining stable. This suggests that there is no evidence, as of yet, for upslope movement of the treeline into the future despite potentially more favourable conditions for their establishment.

L. 326: single not singe

Thank you, we have adjusted this.

**Reviewer #2:**

This is an excellent paper: It is well written, the methods and results are clear, and the authors use the international literature to frame the introduction and provide a nuanced discussion. They build on existing conceptual models, and reframe them for plants with different traits and life history strategies.

The main limitation is that they only have four sites which each have unique grazing and disturbance histories. In otherwords, this study is a comparison of four case studies. While inferences would be stronger with more sites, these case studies are still valuable. The inferences the authors draw from those sites are sound, and are important for predicting how treelines worldwide will shift in response to climate change.

Thank you for raising this and we understand your concerns. Four sites is a limited sample size, however we believe that this is (partly) overcome by revisiting the same four sites almost 20 years apart. Hence, we get an important temporal perspective. In an era of regional warming, coupled with fire, we don’t see expected increases in treeline and, importantly, show that fire may play a crucial role in this dynamic. This intriguing finding suggests much more attention is warranted in understanding the fire-tree-climate interaction at alpine treelines.

A second limitation is that they are inferring that fire is the main cause of demographic changes in tree populations. This may be very reasonable to do, given the dynamics of these ecosystems, but the authors should add some context for international readers: are how do they know that the fires were the main cause of these changes? Can the rule out windthrow, avalanches, insects, etc? The authors need to add text providing this context.

Thank you, we have included a sentence to address this in the methods. In the Victorian Alps we can rule out windthrow, as the trees are not pushed over by strong winds or snow-loading, nor are branches regularly snapped off. Similarly, avalanches are uncommon as the slopes that we work on are not steep enough, nor get enough snow-loading to produce avalanches. Additionally, these sites have not been affected by the *Phoracantha* beetle that is currently in *E. pauciflora* woodlands in Kosciuszko National Park nor are heavily enough affected by other insects to cause mortality. As such we are confident that the changes in demographics we observe are due to these most recent fires.

This study will make an excellent contribution to the global literature on treeline dynamics and wildfire interactions at ecotones.

The authors state the data will be made available but they have not done so yet.

Thank you, our data has now been made available at Harvard Dataverse; <https://doi.org/10.7910/DVN/I5MNND>.

42-64 This is a really nice discussion of resprouting, and the variety of ways resprouting occurs in species and populations. Nice set-up for the whole article.

Thank you.

Figure 1. I think it is great to include a conceptual diagram like this, which allows readers to visualize the dynamics of your study ecosystem. I’m familiar with the other paper you cite, which presents a similar model for obligate-seeding conifer forests. This is a nice modification for resprouting forests. Fig. 1d, is probably the most interesting, with the interaction with climatic stress and vulnerability to repeat fire. You don’t show it, but I can think of treelines where vulnerability to repeat fire may be greatest further away from the climatic limit to growth. In fact, I think the original paper you cite suggests that is the case in North America temperate forests. You might want to make that clear, and consider again (more explicitly) in the discussion how interactions between stress and fire impact immediate ecotone patterns, and long-term change.

Thank you for raising this. We have included a sentence in the discussion acknowledging the importance of understanding how fire and climate will also affect lower elevation woodlands, including the lower montane *E. pauciflora* boundary. We are unable to comment specifically on how this lower montane *E. pauciflora* boundary may respond to these changes as this would require further study, however we have referenced Bowman *et al*. 2014 which reported on the loss of lower elevation obligate seeder mountain forests (*Eucalyptus delegatensis*) due to abrupt fire regime change, which reinforces the potential for substantial shifts in many ecotone boundaries including alpine/subalpine and subalpine/montane.

132: S1 Table 1 shows grazing history as well, which varied greatly between some of the sites. Do you need to mention that history in the text?

Thank you, we have now added a sentence to address grazing history.

132: S1 Fig1: This is an excellent map. I’d suggest including it in the main manuscript. This context is needed for any international readers.

Thank you, we have now included this map in the main manuscript.

149-154: Given the current fires in NSW, you many need to modify lines 149-151 to say “Since 2000 to the time of measurement in 2018…” And then you may need to add a line to note that the pattern of repeat burning is continuing. It looks like “The Twins” site is within the boundary of active MODIS returns of the current fires.
(The extent if the current wildfire impacts in NSW and Victoria are dominating the news worldwide right now. Simply suggesting edits to your text without acknowledging the scale of the ongoing human disaster seemed inappropriate. My condolences if the current fires are impacting people you know, in addition to your research sites.)

Thank you for acknowledging the current fires, we have now included a sentence to acknowledge this in the introduction.

148-149: Disturbance rates should be described using standard frequency measures in fire ecology, such as fire rotation or mean fire return interval, if possible.

Thank you, we have now described disturbance rates in terms of fire return interval.

157-165: Methods are clear and defensible. Good.

Thank you.

169-170: If neither of the two permanent pegs at a transect could not be found, was a transect dropped? or did you re-establish it using GPS coordinates. Either may be fine (depending on the original GPS accuracy), but re-write this sentence to make your re-measurement methods clear.

Thank you for raising this, we have now adjusted the sentence to be clearer.

172-177: Were your field protocols simply confirming—at the tree or transect scale—what was already shown on maps of fire perimeters? If so, make this clear.

Thank you for raising this. Our field surveys identified some inconsistencies with the fire maps. Upon field observations Mt McKay and Mt Feathertop had only been burnt once, as evidenced by the presence of a single burnt cohort on trees. We have included a sentence to address this in the methods and a sentence in Figure 2 caption to acknowledge this inconsistency.

139: Condit et al.’s method was developed to tropical trees, but has been wildly applied. Your use of it seems reasonable given your goals.

Thank you, we agree it has been widely applied and thus its use for sub-alpine woodlands valid.

271: You could also cite Wang et al. 2019 here – you cite it elsewhere.

Thank you, we have included this citation.

301-306: You may want to look at and possibly cite this recent experimental paper on freezing damage. It is from North America, not Australia, but well-controlled experiments like this one are rare.
Maher, C.T., Nelson, C.R. and Larson, A.J., 2019. Winter damage is more important than summer temperature for maintaining the krummholz growth form above alpine treeline. Journal of Ecology.

Thank you for sharing this paper and we appreciate the suggestion, however have chosen not to cite it. For Australian alpine and subalpine areas spring and autumn, rather than winter per se, are the critical periods for freezing stress in seedlings and saplings.

312-322: Good discussion of the lack of upslope migration. This fits well with Harsch et al’s conceptual paper on “treeline forms”—and their description of the limiting factors for “abrupt treelines”—which you cite in the conclusion. I think it would be appropriate to cite here, and perhaps draw in that conceptual framework to this discussion.

Thank you, we have now included a sentence to that effect in the discussion.

344-346: These two lines repeat verbatim information presented in the introduction on lines 122-124. Re-phrase.

Thank you, we have now rephrased this sentence.

Figures: In the .pdf I was provided, it is very hard to read the axis labels and tic labels of the figures, particularly for Figure 2. I was able to make sense of it from the captions, though. The figures may be published in higher resolution, but the font size should also be increased.

Thank you, we have increased the text size within figures and increased resolution.

Figure 3: Could you add vertical dashed lines to this figure, showing the time of disturbance(s) at each site? That type of visual reference would be helpful.

Thank you, we have included vertical dashed lines.

Figure 4: Consider adding the summary statistics as text to each panel in this figure: the data, slope, and what type it is. Then define the types in the figure caption. This would allow the figure to stand alone, and not be reliant on the text.

Thank you, we have included the summary statistics.
